# Impact of Cholesterol Metabolism in Immune Cell Function and Atherosclerosis

**DOI:** 10.3390/nu12072021

**Published:** 2020-07-07

**Authors:** María Aguilar-Ballester, Andrea Herrero-Cervera, Ángela Vinué, Sergio Martínez-Hervás, Herminia González-Navarro

**Affiliations:** 1 INCLIVA Institute of Health Research, 46010 Valencia, Spain; m.aguilar.ballester@gmail.com (M.A.-B.); anhecer@alumni.uv.es (A.H.-C.); m.angela.vinue@uv.es (Á.V.); sergio.martinez@uv.es (S.M.-H.); 2 Endocrinology and Nutrition Department Clinic Hospital and Department of Medicine, University of Valencia, 46010 Valencia, Spain; 3 CIBER de Diabetes y Enfermedades Metabólicas Asociadas (CIBERDEM), 28029 Madrid, Spain; 4 Department of Didactics of Experimental and Social Sciences, University of Valencia, 46010 Valencia, Spain

**Keywords:** cholesterol, inflammation, atherosclerosis, immune cells, hematopoiesis, metabolism

## Abstract

Cholesterol, the most important sterol in mammals, helps maintain plasma membrane fluidity and is a precursor of bile acids, oxysterols, and steroid hormones. Cholesterol in the body is obtained from the diet or can be de novo synthetized. Cholesterol homeostasis is mainly regulated by the liver, where cholesterol is packed in lipoproteins for transport through a tightly regulated process. Changes in circulating lipoprotein cholesterol levels lead to atherosclerosis development, which is initiated by an accumulation of modified lipoproteins in the subendothelial space; this induces significant changes in immune cell differentiation and function. Beyond lesions, cholesterol levels also play important roles in immune cells such as monocyte priming, neutrophil activation, hematopoietic stem cell mobilization, and enhanced T cell production. In addition, changes in cholesterol intracellular metabolic enzymes or transporters in immune cells affect their signaling and phenotype differentiation, which can impact on atherosclerosis development. In this review, we describe the main regulatory pathways and mechanisms of cholesterol metabolism and how these affect immune cell generation, proliferation, activation, and signaling in the context of atherosclerosis.

## 1. Introduction

Cholesterol is the main sterol in mammals and has a key role in the plasma membrane where it is responsible for modulating membrane fluidity, permeability, and signaling [1]. It is also found in the endoplasmic reticulum (ER) membrane in small amounts where it is essential for its metabolic regulation [2]. Cholesterol is also involved in cellular proliferation and embryonic signaling and is a precursor of bile acids, oxysterols, and all steroid hormones.

Cholesterol blood levels are determined by the ingestion of dietary cholesterol and by de novo synthetized cholesterol from acetyl coenzyme A (acetyl-CoA). Cholesterol homeostasis is tightly regulated by the liver, which controls lipoprotein assembling, secretion, and catabolism [1]. Altered cholesterol metabolism and regulation of its circulating levels have a major impact on atherosclerosis, a chronic inflammatory disease. Given that cholesterol is needed for all cellular membranes, genes involved in cholesterol biosynthesis, regulatory pathways, transport, and receptors are ubiquitous across all cell types, including inflammatory and immune cells. Recent studies have indicated that atherosclerosis progression is affected not only by circulating cholesterol levels but also by cholesterol content and metabolism within immune cells, even in the absence of changes in systemic cholesterol levels or defects in cholesterol transport.

In this review, we summarize cholesterol metabolism and transport and the impact of these on immune cell function, production, and activity.

## 2. Cholesterol Metabolism

**Figure 1:**
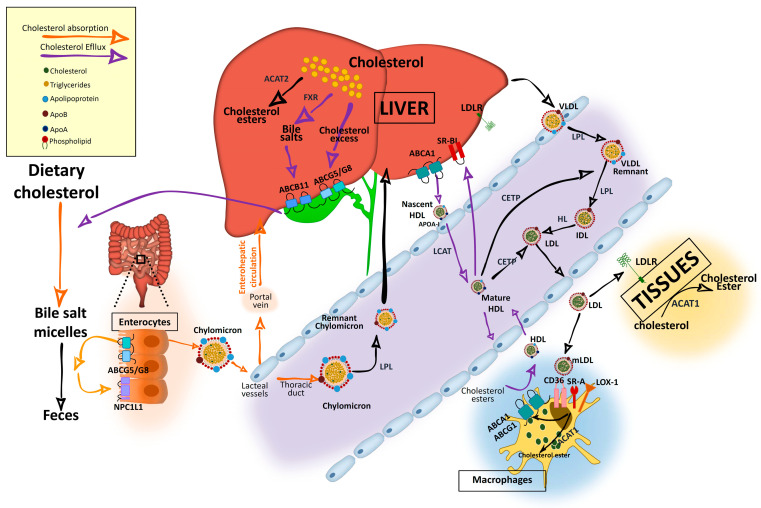
Cholesterol and lipoprotein metabolism.

**Figure 2:**
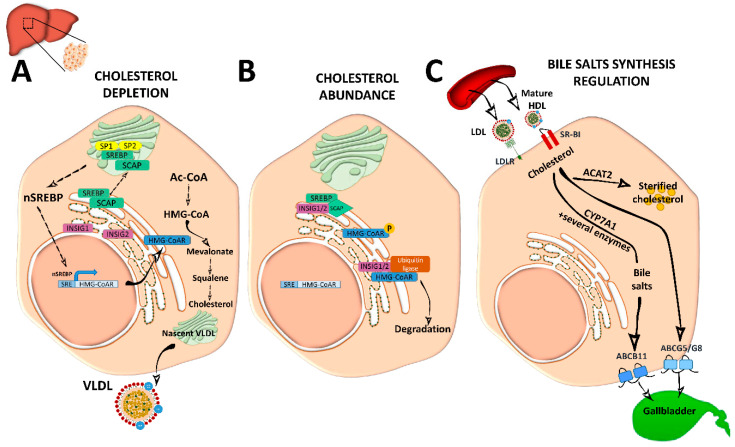
Cholesterol and bile acid biosynthesis. (**A**) In conditions of cholesterol depletion, the sterol regulatory element-binding protein 2 (SREBP2)–SREBP cleavage activating protein (SCAP) complex in the ER is transported to the Golgi apparatus where it is sequentially cleaved by site 1 protease (SP1) and SP2 at different sites to become active. Active nuclear (n)SREBP2 enters into the nucleus and binds to sterol regulatory element (SRE) sequences of target genes to induce the expression of genes involved in cholesterol synthesis such as the rate-limiting cholesterol synthesis enzyme 3-hydroxy-3-methyl-glutaryl coenzyme A reductase (HMG-CoAR). De novo synthetized cholesterol is stored or assembled in very low density lipoproteins (VLDL) in the Golgi apparatus for secretion into the circulation. (**B**) When ER membrane cholesterol exceeds 5% mol of the total lipids, sterol-sensing domains (SSD) in SCAP undergo a conformational change and the complex binds to insulin-induced gene 1 (INSIG1) or INSIG2, remaining inactive. Meanwhile, HMG-CoAR also binds to INSIG1 or INSIG2 for ubiquitilation and degradation, or it is inactivated by phosphorylation. (**C**) Cholesterol enters into the hepatocyte by low-density lipoprotein (LDL) receptor (LDLr) or through scavenger receptor class B type 1 (SRBI) by the delivery of high-density lipoprotein (HDL) reverse cholesterol transport. In the liver, it is stored as cholesterol ester after being esterified by acetyl coenzyme A (Ac-CoA) acetyltransferase 2 (ACAT2). It can also be used to synthetize bile acids in the biosynthetic metabolic pathway with the cytochrome P450 family 7 subfamily A member 1 (CYP7A1) enzyme as a rate-limiting factor. Bile acids will be secreted into the gallbladder as bile salts by adenosine triphosphate-binding cassette subfamily B member 11 (ABCB11) transporter. Cholesterol excess is also effluxed by adenosine triphosphate-binding cassette transporter G5/G8 (ABCG5/G8) into the gallbladder for secretion into the intestine.

In the sections below, we provide an overview of the main and most current pathways and key modulators of intracellular cholesterol metabolism and circulating cholesterol levels, which are also summarized in Figure 1 and Figure 2.

### 2.1. Cholesterol Absorption and Metabolism

In the intestinal lumen, cholesterol binds to bile salt micelles and is transported by Niemann–Pick C1-Like 1 (NPC1L1) through the enterocyte brush border membrane via clathrin-mediated endocytosis [1,2,3] (Figure 1). NPC1L1 is a cholesterol-sensing receptor that traffics from the endocytic recycling compartment to the membrane [3]. During this process, ras-associated binding protein (RAB)11a bound to NPC1L1 is replaced by cell division control protein (CDC)42, which activates actin polymerization promoters that traffic NPC1L1 back to the cell surface. Under cholesterol depletion condition, NPC1L1 is also regulated by sterol regulatory element-binding protein 2 (SREBP2) transcription factor and lysosomal and ubiquitin–proteasome degradation [3,4]. Inside the enterocytes, the cholesterol is re-esterified by acetyl-CoA (Ac-CoA) acetyltransferase 2 (ACAT2) enzyme [5] and assembled into chylomicrons, a triglyceride-rich lipoprotein type with a cholesteryl ester-rich core and a single molecule of apolipoprotein B (ApoB)48 protein on its surface. These nascent chylomicrons are secreted into the intercellular space, migrate to the lamina propria, and enter the lacteal vessels of the lymphatic system for transport through the portal vein into the liver or into the circulation via the thoracic duct [2] (Figure 1).

Dietary cholesterol is digested, solubilized in bile salt micelles in the intestine, and absorbed at the apical region of enterocytes by NPC1L1, where it is assembled in nascent chylomicrons. Chylomicrons will be transported to the liver through the portal vein of the enterohepatic circulation although they can enter into the circulation through the thoracic duct. Cholesterol can also be synthetized endogenously from Ac-CoA. In the liver, it is assembled in very low density lipoproteins (VLDLs), which are secreted into the circulation. Triglycerides in chylomicrons and VLDL are hydrolyzed by lipoprotein lipase (LPL), increasing their density and generating chylomicron remnants and low-density lipoprotein (LDL) particles that can be further catabolized by hepatic lipase (HL). VLDL and LDL are also enriched in cholesterol by cholesteryl ester transfer protein (CETP), which transfers cholesteryl esters from high-density lipoprotein (HDL). Resultant chylomicron remnants are taken up by the liver and the components enter into the lipoprotein packaging. LDL delivers cholesterol by an LDL receptor (LDLr)-mediated endocytic process to most of the tissues. In the subendothelial space, LDL undergoes a variety of modifications, such as oxidation, acetylation, or aggregation to generate modified LDL (mLDL), which is taken up by macrophages through scavenger receptor (SR)-A, cluster of differentiation (CD)36, and low-density lipoprotein receptor-1 (LOX-1) to become foam cells. The excess of cholesterol undergo an efflux process mediated by adenosine triphosphate-binding cassette transporter A1 (ABCA1) and adenosine triphosphate-binding cassette transporter G1 (ABCG1), which interact with apoliproprotein A (ApoA)-I of the cholesterol acceptor nascent discoidal lipid-poor HDL. Mature spherical lipid-rich HDL returns by the reverse cholesterol transport to the liver where it delivers cholesterol through scavenger receptor class B type 1 (SRBI). The liver stores cholesterol that can be used to synthetize bile salts in a farnesoid X receptor (FXR)-regulated pathway with the rate-limiting enzyme cytochrome P450 family 7 subfamily A member 1 (CYP7A1). Cholesterol can also be secreted into the gallbladder by adenosine triphosphate-binding cassette transporter G5/G8 (ABCG5/G8) along with bile salts, which are secreted by adenosine triphosphate-binding cassette subfamily B member 11 (ABCB11) until they are secreted in the intestine for elimination with the feces. Reabsorption by NPC1L1 at the apical side of enterocytes can incorporate cholesterol back into chylomicrons. For intracellular storage, cholesterol is esterified by ACAT2 in hepatocytes and by ACAT1 in the rest of the cell types.

### 2.2. Endogenous Synthesis of Cholesterol

Cellular depletion of cholesterol activates endogenous cholesterol biosynthesis from Ac-CoA molecules, in an energetically expensive anabolic pathway regulated by SREBP2 and the 3-hydroxy-3-methyl-glutaryl coenzyme A reductase (HMG-CoAR). SREBP2 is synthetized as an ER-anchored precursor that is translocated with the SREBP cleavage activating protein (SCAP) to the Golgi apparatus for activation through cleavage by site 1 protease (SP1) and SP2. Insulin-induced gene 1 (INSIG1) and INSIG2 proteins retain SREBP–SCAP dimer interaction when SCAP sterol-sensing domains (SSD) detect ER membrane cholesterol over 5 mol % of total lipids [3,4,6,7] (Figure 2).

Activated nuclear SREBP binds to sterol regulatory element (SRE) sequences to activate transcription in target genes including its own; its synergistic inductors nuclear transcription factor Y subunit alpha (NF-Y) and SP1; its inhibitor FOXO3; and HMG-CoAR, an ER-glycoprotein that converts the HMG-CoA to mevalonate and is inhibited by non-sterol isoprenoids [3]. Cholesterol abundance diminishes HMG-CoAR activity by INSIG1 and INSIG2, which bind to HMG-CoAR with ubiquitin ligases for degradation through different pathways [8] (Figure 2). Oxysterols; methylated sterols; geranylgeraniol, an intermediate of vitamin biosynthesis; and two vitamin family members (δ- and γ-tocotrienol) are also strong inducers of HMG-CoAR degradation. HMG-CoAR can reversibly be inhibited by phosphorylation [3,4] (Figure 2).

Hepatic de novo synthesized cholesterol is assembled in the Golgi apparatus in very low density lipoprotein (VLDL) particles, which are triglyceride-rich and cholesteryl ester-poor lipoproteins with a single molecule of ApoB100 on its surface [2,5], and are secreted into the circulation to be distributed among the other tissues (Figure 2).

### 2.3. Cholesterol Transport and Lipoprotein Metabolism

In the bloodstream, triglycerides in chylomicrons, very low density chylomicrons, and VLDL are hydrolyzed by lipoprotein lipase (LPL) enzyme, present in the endothelial surface of capillaries of most tissues and will be used as a source of energy or stored intracellularly [2]. Cholesteryl ester-rich chylomicron remnants are taken up by the liver and its components are incorporated in the newly synthetized VLDL particles [1]. VLDL will become first intermediate-density lipoproteins (IDL) and via hepatic lipase (HL) action will generate low-density lipoproteins (LDL). These lipoproteins can be further catabolized by lipases, a process that increases their density. In addition, VLDL and LDL can also be enriched in cholesterol by the cholesteryl ester transfer protein (CETP), which transfers cholesteryl esters from high-density lipoprotein (HDL) [2]. Resultant LDL are cleared by the LDL receptor (LDLr) in different tissues [9] (Figure 1). LDL-bound LDLr undergoes the clathrin-mediated endocytosis and cholesteryl esters will enter into the endolysosomal system to transport cholesterol to the ER. LDLr returns to the plasma membrane by an endosomal recycling complex or undergoes lysosomal degradation through binding to proprotein convertase proprotein convertase subtilisin kexin 9 (PCSK9), which is strongly upregulated by SREBP and downregulated by insulin/mammalian target of rapamycin (mTOR)c signaling [3]. LDLr can also enter in lysosomal degradation by polyubiquitylation-inducible degrader of LDLr (IDOL) E3 ligase (also called MYLIP).

### 2.4. Cellular Cholesterol Efflux and Storage

Mature spherical lipid-rich HDL returns by the reverse cholesterol transport to the liver where it delivers cholesterol through the SRBI for excretion (Figure 1) [4]. HDLs are the densest and smallest particles with a high protein content of mostly ApoA-I protein. ABCA1 and lipid-free ApoA-I are responsible for removing cholesterol excess from cells and form nascent discoidal HDL particles that contain ApoA-I. Once released into the circulation, nascent HDL becomes spherical mature HDL by LCAT, CETP, and HL action [3,9] (Figure 1). ApoA-I can also be endocyted in clathrin-coated vesicles and directly receive LDL-derived cholesterol from late endosomes via Niemann-Pick type c (NPC)2. Notably, in macrophages, ABCA1 and ABCG1 have a major role in cholesterol removal by preventing foam cell formation. ABCA1 and ABCG1 are upregulated by retinoid X receptor (RXR) and liver X receptor (LXR). Transactivation of ABCA1 is also upregulated by LXR-responsive long non-coding RNA (lncRNA) called MeXis [3]. Back in the liver, HDL-derived sterols bind to taurine, glycine, or sulfate, generating bile salts that are secreted by ABCB11 transporter to the canalicular membrane to form bile salt-phosphatidylcholine micelles [1,3] (Figure 2). This HDL-mediated pathway is key in cholesterol metabolism as it is the main pathway of cholesterol removal from the body. Hepatic ABCG5 and ABCG8, a heterodimeric transporter (ABCG5/G8) positively regulated by LXR, excretes excess neutral sterol into the bile [1,3,10] (Figure 1 and Figure 2). After food ingestion, bile stored in the gallbladder is secreted into the intestine where it is reabsorbed by membrane protein NPC1L1 or excreted with the feces. In the liver, bile acids also act as ligands of several nuclear receptors such as FXR and pregnane X receptor (PXR) [1]. FXR also functions as a bile acid sensor, regulating bile acid synthesis genes that inhibit CYP7A1, a rate-limiting enzyme in bile acid synthesis [1,10,11] (Figure 2).

Intracellular cholesterol is esterified by two membrane enzymes, ACAT1 and ACAT2 [3]. ACAT1, found predominantly in macrophages, epithelial cells, and steroid hormone-producing cells, is activated by cholesterol and by two promoters, P1 and P7 [3]. In enterocytes and hepatocytes, ACAT2 is potently activated by cholesterol and has a greater affinity for 25-hydroxycholesterol and bile acid derivatives. ACAT2 gene expression is regulated by several transcription factors, including hepatocyte nuclear factor 1α (HNF1α), HNF4α and caudal type homeobox 2 (CDX2), and by proteasomal degradation through ubiquitylation [3] (Figure 1 and Figure 2).

## 3. Cholesterol in Atherosclerosis

Altered cholesterol metabolism leads to dyslipidemia and premature atherosclerosis, a process modulated by innate and adaptive immunity [12]. An important event preceding atherosclerosis lesion formation is the accumulation of ApoB-containing lipoproteins, mostly LDL, in the subendothelial space [12,13]. These lipoproteins are susceptible to modification by oxidation, acetylation, and aggregation, which enhances their proinflammatory actions [14]. On the other hand, endothelial dysfunction is a major determinant of lipoprotein retention, especially important in vascular bed regions with high shear stress force, as well as in several conditions such as aging, low-grade inflammation, or oxidative stress produced by other pathologic conditions such as hypertension or diabetes [14,15]. In the initial stages, lipoprotein accumulation worsens endothelial dysfunction, which is characterized by expression of adhesion molecules and permeability of the endothelial layer. These changes lead to increased adhesion, retention, and migration of immune cells in the subendothelial space [12,15].

Retained monocytes differentiate into macrophages, acquiring either an anti-inflammatory phenotype, which contributes to a more stable and fibrotic plaque, or a pro-inflammatory phenotype, uptaking ApoB-containing lipoproteins to become foam cells that, when accumulated, form fatty streak lesions [12,15]. Macrophages at this stage are modulated by modified LDLs, which are described in more detail in Section 4.2. Briefly, inflammasome (NLRP3) activation; increased secretion of cytokines such as interleukin (IL)1β, IL6, and tumor necrosis factor (TNF)α; and reactive oxidant species (ROS) production sustains atheroma inflammation [12,13,15]. Nonetheless, ABCA1- and ABCG1-mediated cholesterol efflux pathways into lipid-poor HDL, activated by sterol-regulated LXR transcription factors in macrophages, ameliorate atheroma formation and inhibit NLRP3 activation [14]. When lesions progress to medium stage, they affect vascular smooth muscle cells (VSMCs), which transdifferentiate to diverse phenotypes such as lipid-loaded macrophage-like cells with a proinflammatory, migratory, and proliferative phenotype. VSMCs might also acquire myofibroblast characteristics, which together with the extracellular matrix form a fibrous cap above a lipid core made up of lipid-loaded macrophages [12,16]. Monocytes can also differentiate into dendritic cells (DCs) contributing to cytokine signaling, regulatory T (Treg) cell repression, and antigen-presenting stimulation to effector T helper (Th) cells [13,14]. During lesion development, the balance of different T cell subsets determines the generation of obstructive vulnerable plaques. Th1 and Th17 cells are pro-inflammatory and proatherogenic, while Th2 cells secrete anti-inflammatory cytokines and Treg cells suppress CD4+Th and cytotoxic CD8+T cell activity and promote anti-inflammatory macrophage phenotype and inflammatory resolution [14,17]. Decreased Treg/Th17 cell ratio is consistently associated with chronic inflammation, atherosclerosis progression, and vulnerable plaque syndromes [18,19,20]. At later stages, the unbalanced ratio of immune cells leads to unresolved chronic inflammatory state, generating clinically unstable plaques characterized by large necrotic cores, due to VSMC and macrophage death, covered by thin fibrous caps [12,21].

## 4. Cholesterol Metabolism in Immune Cells

**Figure 3:**
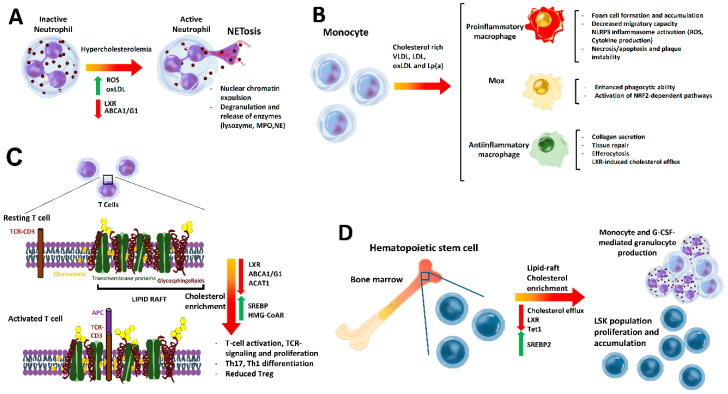
Impact of cholesterol on immune cells. (**A**) Neutrophil activation is induced under hypercholesterolemic conditions, in which oxidized (ox)LDL and reactive oxidant species (ROS) levels increase, while cholesterol efflux genes such as liver X receptor (LXR) and transporters adenosine triphosphate-binding cassette transporter A1 (ABCA1) and adenosine triphosphate-binding cassette transporter G1 (ABCG1) are downregulated. Activated neutrophils contribute to atherosclerotic plaque formation and instability by NETosis, a process consisting of the release of neutrophil extracellular traps (NETs) and secretion of lysozyme, myeloperoxidase (MPO), and neutrophil elastase (NE), which contributes to endothelial injury. (**B**) Hypercholesterolemia induces macrophage activation, differentiation, and function in atheroma lesions. Monocyte-derived macrophages accumulate cholesterol and form foam cells through the uptake of lipoproteins (chylomicron remnants, VLDL, LDL, oxLDL, and apolipoprotein (a) (Lp(a)), thus forming atherosclerotic plaque. In the atheroma plaque environment, macrophages acquire a proinflammatory phenotype as intracellular cholesterol accumulation activates inflammasome (NLRP3), enhancing the production of cytokines and ROS. ApoB lipoproteins might induce apoptosis and necrosis of stressed macrophages, facilitating the generation of vulnerable plaques. Anti-inflammatory macrophages, with a proresolving phenotype, secrete collagen; induce tissue repair; and enhance macrophage cholesterol efflux, efferocytosis, and reverse cholesterol transport. A third type of macrophage called M(ox) has been described, which displays decreased phagocytic activity and promotes the overexpression of genes controlled by the transcription nuclear factor erythroid 2–related factor (NRF)2. (**C**) Enrichment of cholesterol in lipid rafts in T cells facilitates the clustering of T cell receptor (TCR) signaling complexes and hence immune synapse. These processes lead to T cell activation and proliferation, T helper (Th)17 and Th1 differentiation, and a decrease in the regulatory T (Treg) population. To ensure T cell expansion, SREBP- and HMG-CoAR-mediated cholesterol synthesis is also upregulated, while LXR-dependent efflux pathways are repressed. (**D**) Cholesterol enrichment of lipid rafts in hematopoietic progenitor/stem cells (HPSC), caused by hypercholesterolemia and/or a defective cholesterol efflux, affects HPSC quiescence, proliferation, migration, and hematopoiesis. Activated SREBP2-signaling increases cholesterol levels and affects HPSC differentiation. Decreased LXR-dependent cholesterol efflux promotes proliferation of Lin-Sca+cKit+ (LSK+) HPSC population and granulocyte-colony stimulating factor (G-CSF)-mediated granulocyte production, enhancing circulating monocytes and neutrophils. High cholesterol levels also downregulate *TET-1* gene expression in HPSC, which impairs its stem cell capacity.

Cholesterol is needed for all cellular membranes, making its biosynthesis and regulatory pathways ubiquitous across cell types, including immune cells. Recent studies have underlined an emerging role for cholesterol as an important modulator of innate and adaptive immunity activity. In the following sections, we outline the principal findings on cholesterol and how changes in its intracellular metabolism affect immune cell activation and function, which are summarized in Figure 3.

### 4.1. Cholesterol Effect on Neutrophil Biology in Atherosclerosis

Neutrophils are the most abundant circulating white blood cells in humans, terminally differentiated in the bone marrow (BM). With a circulating lifespan of under 24 h, BM produces 10^11^ neutrophils daily, a process regulated by granulopoiesis, mobilization from the BM, apoptosis, and clearance. As the first line of defense, neutrophils are rapidly recruited into acute inflammatory sites in damaged tissues. In these sites, neutrophils display several mechanisms to eliminate pathogens, which include discharge of their granule proteins, alarmins (cathelicidin) and proteases, cathepsin G, neutrophil elastase (NE), and myeloperoxidase (MPO), as well as production of ROS and bioactive lipid mediators [22,23]. Neutrophils also produce neutrophil extracellular traps (NETs), which are net-like DNA complex structures with NE and antimicrobial molecules such as histones [24]. In a process called NETosis, NETs induce endothelial injury, thus playing a crucial role in atherosclerosis [22].

Neutrophil activity has been shown to be modulated by circulating cholesterol levels. Hypercholesterolemia in apolipoprotein E (*ApoE*)*-/-* mice, aggravated by high-fat diet (HFD), produced neutrophilia associated with enhanced lesion size. Neutrophilia enhanced circulating neutrophils by activating G-CSF-induced granulopoiesis and BM mobilization in response to C-X-C motif chemokine ligand (CXCL)1/ C-X-C motif chemokine receptor (CXCR)2 axis activation. High cholesterol levels decreased BM-derived CXCL12 levels which reduced neutrophil clearance. Interestingly, C-C motif chemokine receptor (CCR)1- and CCR5-mediated adhesion, and extravasation of neutrophils were also induced by C-C motif chemokine ligand (CCL)5 platelet secretion [25].

Cholesterol accumulation produced by Western diet feeding in myeloid-deficient *Abca1/g1*-deficient mice increased the NETosis of accumulated neutrophils in incipient atheroma [26]. In another study, HFD-fed *Abcg1-/-* and *Abca1-/-Abcg1-/-* mice displayed G-CSF-induced neutrophilia, which was attributed to facilitated toll-like receptor (TLR)4/ myeloid differentiation primary response (MyD)88 signalling and cytokine production in macrophages caused by cholesterol plasma membrane accumulation [27].

LXRs might also negatively regulate neutrophil homeostasis through inhibition of IL23/IL17 granulopoietic cytokine axis, which increases neutrophil clearance [28]. Consistently, Hong et al. reported that *Lxrαβ-/-* mice presented neutrophilia in peripheral blood, spleen, and liver due to impaired clearance. Accordingly, LXR agonist treatment of a mouse model of sterile peritonitis showed defective neutrophil recruitment by a *cis*-repression of chemokines and adhesion molecule expression [29]. In contrast, human neutrophils treated with oxysterols, 7-keto-cholesterol, or 25-hydroxycholesterol showed increased reduced nicotinamide adenine dinucleotide phosphate (NADPH) oxidase activation, enhanced ROS production, and lysozyme release from neutrophils granules [30].

Cholesterol packed in oxLDL [31] or cholesterol crystals [32] also induced NET formation. oxLDL stimulated ROS-dependent NETosis in human primary neutrophils via a TLR2/6 and protein kinase C (PKC)- interleukin-1 receptor-associated kinase (IRAK)- mitogen-activated protein kinase (MAPK) pathway [31]. Likewise, neutrophils in vitro treated with cholesterol crystals exhibit NETosis and ROS production and elastase translocation to the nucleus. In vivo, hypercholesterolemic *ApoE-/-* mice display NETs in atheroma that primes macrophages to secrete IL1β, thus activating proinflammatory Th17 cells.

Consistent with the above findings, changes in neutrophil activity in hypercholesterolemia leads to lesion size changes. Thus, deficiency in NE and protein 3 in hypercholesterolemic *ApoE-/-* mice reduces lesion size [32]. Likewise, NE deficiency or inhibition in hypercholesterolemic HFD-fed *ApoE-/-* mice diminished neutrophil atheroma content and produced more stable plaques [33]. In another study, deficiency of the neutrophil cathepsin G (*CtsG-/-*) increased atheroma plaque along with cholesterol levels in Western diet-fed *LDLRlr-/-* mice. In agreement with this, reduced CtsG plasma levels have been observed in patients with coronary heart disease and enhanced total cholesterol levels [34].

In humans, hyperlipidemia severity in patients (c-LDL >120 mg/dL) is associated with prevalence of polymorphonuclear leukocytes including neutrophils, eosinophils, and basophils. This was associated with elevated serum levels of MPO and inflammatory markers, fibrinogen, and c-reactive protein (CRP) compared to the control group [35]. Likewise, Puntoni et al. reported that patients with familial hypercholesterolemia had higher MPO serum levels, which lowered when total cholesterol diminished [36]. The neutrophil/lymphocyte count ratio is a consistent biomarker for the prediction of cardiovascular risk and is elevated in patients with low HDL cholesterol compared with the control group [37].

### 4.2. Cholesterol Impact on Monocytes and Macrophages

Hypercholesterolemia affects monocytosis and macrophage activation, differentiation, and function in atheroma lesions [38]. In lesions, monocyte-derived macrophages will form foam cells in the presence of ApoB-lipoproteins. These accumulate cholesterol by VLDL and chylomicron remnant uptake, or more commonly through receptor-mediated modified LDL uptake [39]. Modified LDL and oxLDL bind to CD36 on the macrophage surface and cholesterol is stored as cholesterol crystals in lipid droplets, which induce lysosomal damage [14]. OxLDL can also be ingested via type A scavenger receptor (SRA) and low-density lipoprotein receptor-1 (LOX-1), thus promoting foam cell formation [12,13,15]. Oxysterols and oxidized phospholipids contained in modified lipoproteins, oxLDL, or the apolipoprotein (a) (Lp(a)), an LDL with an additional specific apolipoprotein (a) attached by a single thioester bond to apolipoprotein B-100, can trigger apoptosis of stressed macrophages through activation of TLR [12,40]. Internalized oxLDL has been demonstrated as a ligand of peroxisome proliferator-activated receptor (PPAR)γ and thus enhances CD36 expression itself [41], while activation of TLRs by their ligands enhances lipid uptake by SR expression, contributing to foam cell formation [13]. These later events therefore perpetuate macrophage foam cell formation, also exhibiting decreased migratory capacity. Intracellular cholesterol within macrophages induces NLRP3 inflammasome activation and enhances the production of cytokines such as IL1β, IL6, and TNFα and ROS [12,13,15,39]. Interestingly, the inflammasome can also be activated by epigenetic changes during monocyte differentiation, in a process called priming. Pro-inflammatory macrophages activated by IFNγ might also promote necrosis and thinning of the fibrous cap due to their characteristic presence in different regions prone to rupture. These activated macrophages secrete cytokines and chemokines that enhance inflammation and secretion of different metalloproteinases (MMPs), which contribute to plaque destabilization by degradation of the fibrous cap [41].

Macrophages can also acquire an anti-inflammatory proresolving phenotype, partially induced by IL4 secretion. Mostly present in stable plaques, they secrete collagen to form the fibrous cap, as well as proteins and lipids to promote tissue repair [12,42]. These macrophages also are involved in suppression, regression of atheroma plaque, and efferocytosis [12]. Thus, sterol-regulated transcription factors LXRa and LXRb (LXR) induce ABCA1 and ABCG1 cholesterol efflux into lipid-poor HDL, which has a major role in plaque regression through promotion of reverse cholesterol transport. HDL particle formation in the plaque environment is also important for enzymes such as paraoxonase 1 (PON1), as these prevent LDL oxidation [43]. Nevertheless, accumulation of cholesteryl ester droplets causes a defect in sterol esterification or intracellular cholesterol traffic in some lesional macrophages, which impairs cholesterol efflux [12,14]. Besides cholesterol efflux, LXR can suppress inflammation by inhibiting TLR-dependent signaling, while TLR activation suppresses LXR activity [38,44,45]. Specifically, LXR small ubiquitin-like modifiers (SUMO)ylation binds to nuclear factor kappa B (NF-κB), preventing transcription of their target genes. Therefore, BM-derived macrophages (BMDM) treated with LXR agonist and LPS demonstrated repression of NF-κB target genes [45]. Furthermore, LXR promotes the synthesis of long-chain polyunsaturated fatty acids (PUFA), which induce inflammation resolution through synthesis of eicosanoids and proresolving lipid mediators.

LXR also induces expression of tyrosine protein kinase MER (MERTK), which leads to efferocytosis. This process involves clearance of apoptotic cells by macrophages, which in turn upregulate ABCA1 and ABCG1 expression, reducing the TLR4-signalling pathway. Thus, efferocytosis of apoptotic macrophages is key for reducing plaque necrosis and inflammation, and involves robust efflux of cholesterol and pro-apoptotic oxidized lipids. However, certain mechanisms might contribute to defective efferocytosis, such as defective cholesterol efflux, lipoprotein-associated hydrolysis of oxidized phosphatidylserine on the surface of apoptotic cells by phospholipase A2 (PLA2), or changes in the plasma membrane structure of macrophages due to saturated fatty acid exposure [12].

A third macrophage type named M(ox) has been detected, which has less migratory and phagocytic ability than the other two subtypes, and which overexpresses genes controlled by transcription factor NRF2 [42].

Although macrophages are modulated in situ within lesions by cholesterol, circulating monocytes can also be influenced by hypercholesterolemic conditions. Thus, hypercholesterolemic *ApoE-/-* mice fed a Western diet display increased monocyte production or monocytosis. Moreover, in these conditions, monocytes display Ly6C^hi^ monocytes, a proinflammatory phenotype with enhanced Ly6C expression, which are invasive and associated with plaque progression. Ly6C^hi^ monocytes also exhibited a longer lifespan in circulation with an impaired switch to proresolving and anti-inflammatory Ly6C^low^ phenotype [46]. Another study also observed that HFD in *ApoE-/-* mice increased activated CD11c+ monocyte levels compared to mice fed a normal diet [47]. Hence, BM monocytes with a proinflammatory and proinvasive phenotype produce hypercholesterolemia, which promotes atherogenesis. In humans, children with familial hypercholesterolemia and reduced HDL levels also exhibit elevated monocyte numbers compared to control group [48].

Before being recruited to the artery wall, monocytes can also be primed toward a long-lasting pro-inflammatory phenotype upon exposure to oxLDL [42,44]. Consistent with this, priming monocytes from atherosclerotic patients with LPS and IFNγ enhanced secretion of IL6 and IL1β compared with monocytes from healthy individuals. Interestingly, this phenotype was maintained during macrophage differentiation, suggesting that atherosclerotic patient monocytes have a pro-inflammatory phenotype [49]. Finally, oxLDL and hypercholesterolemia induce innate immune memory, a process in which monocytes remodel the chromatin to create a response to a previously recognized stimulus. It has been reported that human monocytes exposed to oxLDL and then treated with TLR agonists or LPS present an inflammatory response [42].

### 4.3. Cholesterol in T Cell Function and Phenotype

T cell activation and proliferation has been shown to require cholesterol or derivatives to build up membranes and act as signals to promote cellular events such as differentiation, growth, and gene expression by binding to different transcription factors.

Changes in T cell cholesterol content affect lipid rafts, which are highly ordered cholesterol- and ganglioside-rich membrane lipid microdomains.

In resting T cells, T cell receptor (TCR) and CD3 reside outside of rafts [50], but cholesterol enrichment of lipid rafts cluster together diverse components of TCR signaling complexes. This facilitates the immunological synapse that forms between antigen-presenting cells (APC) and TCR, as well as activation of downstream pathways. Conversely, disordered lipid rafts in T cells produce more unstable immune synapses and TCR signaling [12]. Cholesterol content in lipid rafts is in this way a checkpoint for T cell activation and function and immunological synapse. Interestingly, the hyperactivity and hypersensitivity of T cells observed in autoimmune diseases, such as systemic lupus erythematosus or rheumatoid arthritis, is associated with membrane cholesterol enrichment in lipid rafts and facilitates TCR-dependent signaling [12]. Likewise, it is presumed that metabolic alterations that accelerate atherosclerosis are partly due to changes in lipid raft dynamics and colocalization of receptors modulating T cell responses and phenotypes.

Moreover, a number of studies have underlined the critical need for balanced cholesterol metabolism in T cells to maintain lipid raft homeostasis for proper T cell function and signaling. Hence, impairment in cholesterol metabolic enzymes, cholesterol modulators, and transporters lead to changes in T cell function, activation, and reprogramming.

Immune synapse formation robustly induces HMG-CoAR cholesterol synthesis for membrane biogenesis and proliferating T cells while repressing LXR-dependent efflux pathways. Consequently, LXR ligation was found to impair mitogenic signals in T cells by an ABCG1-dependent mechanism, while LXRβ inactivation promoted proliferation and lymphoid hyperplasia [41]. In vitro studies have shown that LXR stimulation impaired Th1 and Th17 cytokine production and promoted Treg cell by decreasing lipid raft cholesterol content through enhanced cholesterol efflux [51]. Other studies have also shown different PPARs as involved in T cell regulation—activation of PPARγ and PPARα agonists impairs T cell proliferation, PPARγ might inhibit Th differentiation [52], while PPARδ stimulation promotes T cell proliferation by an extracellular receptor kinase (ERK)-dependent mechanism and enhances Th1 and Th17 responses [51]. Cytotoxic effector CD8+T cells requires SREBP proteins to provide sufficient cholesterol levels to allow membrane biogenesis for blastogenesis and clonal expansion, and to acquire an effector phenotype [53].

Circulating cholesterol levels also affect T cell activation and reprogramming. Western diet-induced hypercholesterolemia in mice was found to promote CD4+T cell differentiation in the liver [54]. Hypercholesterolemia in mice enhanced intrahepatic Foxp3+Treg and RAR-related orphan receptor (ROR)γTh17 cells and tumor growth factor (TGF)β, which is determinant for their differentiation. Interestingly, in *LDLR*-deficient mice, with activated cholesterol biosynthesis, Th17 remains unchanged but intrahepatic Th1 cell differentiation is enhanced. This could indicate that endogenous newly synthetized cholesterol affects Th1 differentiation, while exogenous levels from a cholesterol-enriched diet instead affect Treg and Th17 production. Notably, adoptive transfer analysis also demonstrated that T cell differentiation was directly affected by liver-derived antigens induced by hypercholesterolemia rather than by the inflammatory milieu of the atheroma, as transferred hepatic differentiated CD4+ T cells homed to atheroma and prevailed against endogenous splenic, thymic, or lymphoid T cells [54]. These aforementioned studies indicate that hypercholesterolemia affects circulating and hepatic T cell phenotype and differentiation. In other research, circulating hypercholesterolemia in humanized mice, induced by adeno-associated virus 8–PCSK9 treatment and Western diet, resulted in disrupted human T cell homeostasis with enhanced blood, and hepatic and lung CD4+ and CD8+ memory T cells, while Treg cells were decreased [55].

Various other studies indicate that manipulation of membrane cholesterol content through different means directly affects T cell function. Enrichment of membrane cholesterol through squalene administration in mice augmented peripheral CD4+T cell content, promoted Th1 differentiation by colocalization of IL2Rα, IL4Rα, and IL12Rβ2 subunits within lipid rafts; and enhanced signal transducer and activator of transcription (STAT)4 and STAT5 phosphorylation [56]. This cholesterol enrichment did not, however, affect the suppressive functions of CD4+Foxp3+ Treg cells [56]. This research suggests that cholesterol-enriched membranes produced by various physiopathological conditions such as hypercholesterolemia may bias the immune system toward an inflammatory Th1 type response. In another study, cholesterol enrichment through T cell-specific ABCG1 absence, which prevented cholesterol efflux, led to increased cholesterol content and dynamics in lipid rafts. Consequently, these changes augmented TCR signaling and proliferation through increased phosphorylation of the zeta-chain of T cell receptor associated protein kinase (ZAP)70/ERK1/2 TCR proliferation pathway [57]. Likewise, intracellular cholesterol accumulation induced by ABCG1 absence impaired the mTOR pathway and promoted Treg cell differentiation through STAT5 activation, which ameliorated atherosclerosis in *LDLR-/-* mice [58].

In line with the findings above, lowered cholesterol levels in T cells has a profound effect on effector T cells and ameliorates atherosclerosis. Delipidated cell cultures diminished cholesterol content and lipid raft organization, and consequently impaired TCR-induced proliferative response in CD4+ and CD8+T cells [59]. Similarly, ACAT1 inhibition with the specific inhibitor avasimibe prevented cholesterol esterification, enhanced cholesterol in lipid rafts, and promoted CD8+T cell proliferation through TCR clustering. Avasimibe also improved immunological synapse and cytotoxic activity in these cells [60]. Interestingly, a drop in LDL and HDL cholesterol levels compromised T cell activation, while dietary cholesterol increased the levels of intrahepatic Treg, which migrated to atherosclerotic sites to exert atheroprotection. However, in the presence of hepatic inflammation, intrahepatic Treg differentiation was impaired in favor of proatherogenic Th1 [54]. Of note, in T cells from mice deficient in ApoA1, and hence with low HDL levels, T cell activation was compromised [61]. Consistent with a role of cholesterol accumulation in T cell effector differentiation, HMG-CoAR inhibition by statins, which impaired cholesterol biosynthesis, increased Treg cell differentiation [62]. Given that IL6 signaling through STAT3 inhibits Foxp3 expression in normal conditions, potential inhibition of IL6 signaling by statins could promote Foxp3 expression and increased Treg differentiation [63].

Cholesterol can also modulate TCR signaling via promotion of various conformational states of membrane receptors [64]. It has been shown to modulate TCR clustering and increase interaction with antigens through direct TCR-β binding [65], although in some cases TCR activation might be prevented by cholesterol binding in resting state [66]. Likewise, cholesterol sulfate inhibits TCR signaling through disruption of TCR multimers and by displacing bound cholesterol [67].

Other studies have also found a role for cholesterol derivatives in T cell function. 7β,27-Dihydroxycholesterol was shown to be a selective activator of RORγ, the master nuclear transcription factor needed for Th17 differentiation, and changes in the enzymes related with this oxysterol affected Th17 [68,69]. In another study, Th17 differentiation induced by RORγ was preceded by suppression of cholesterol efflux programs, enhanced cholesterol biosynthesis, and intracellular accumulation of desmosterol [70].

### 4.4. Effect of Cholesterol on Hematopoiesis

Leukocytosis was long ago identified as a nonspecific transient marker of acute ischemic events in cardiovascular disease (CVD) but is currently well known as a potential characteristic of chronic atherosclerosis [71]. Recent research showed that changes in cholesterol transport and metabolic production have a major impact in HPSC quiescence, proliferation, and migration [72], thus affecting leukocytosis. This is due to their response to cholesterol levels, as HPSC express high levels of receptors for lipoprotein uptake—*LDLR, Srb1, ApoE*, and *Lrp1* [48]—and cholesterol-efflux—ABCA1 and ABCG1 [73]. Notably, several studies have demonstrated that defective cholesterol efflux in HPSC leads to cholesterol accumulation in lipid rafts, facilitating HPSC signaling, proliferation, and mobilization.

Hypercholesterolemia induced by Western diet feeding in *ApoE-/-* or *LDLR-/-* mice increased HPSC and leukocyte count and accelerated atherosclerosis [74]. ABCA1 and ABCG1 deficiencies in the BM prevented cholesterol efflux from HPSC, resulting in enhanced proliferation, mobilization, and G-CSF-mediated granulocyte production [73]. Given that these changes were reversed by HDL infusion, the increased mobilization could be attributed to intracellular cholesterol accumulation. These genetic deficiencies also promoted extramedullary hematopoiesis instead of production in the hematopoietic vascular niche of the BM, which pointed to a disrupted BM homeostasis and microenvironment [73]. Along the same lines, tissue-specific double deficient ABCA1/ABCG1 mice in the BM enhanced proliferation of Lin-Sca+cKit+ (LSK+) HPSC population, which displayed cholesterol-enriched lipid rafts and enhanced ERK/(resistance to audiogenic seizures) RAS-dependent activation. Consistently, treatment with lipid raft-disrupting drug cyclodextrin diminished HPSC proliferation [75]. Furthermore, atherosclerosis was decreased in ApoA1 transgenic mice that had facilitated efflux of HDL [75]. In agreement with the above studies, hypercholesterolemic *LDLR-/-* mice also displayed augmented HPSC [76]. Moreover, LSK+HSPC cells treated with LDL induced differentiation toward monocytes and granulocytes through an ERK-dependent mechanism, which was prevented by HDL treatment. Similarly, treatment of mice with human ApoA-I or reconstituted HDL diminished proliferation of HPSC in BM [76].

ApoE also modulates HPSC proliferation, affecting leukocyte accumulation in atheroma. HPSC-derived ApoE binds to cell surface proteoglycans, cooperating with ABCA1/ABCG1-mediated cholesterol efflux [74]. *ApoE* deficiency led to lipid raft cholesterol accumulation, ERK1/2-STAT5 activation, and cell survival and proliferation. Interestingly enough, treating hypercholesterolemic *ApoE*-/- mice fed with an LXR activator reduced the number and proliferation of HPSCs through facilitated HDL efflux. In another study, SRBI deficiency in HPSC resulted in defective HDL-mediated efflux, enhanced HPSC proliferation, and accelerated atherosclerosis [77]. Collectively, these findings indicate that proliferation and hematopoiesis are affected by cholesterol enrichment of lipid rafts, probably by facilitating signaling pathways of HPSC cell growth and differentiation.

The importance of HDL and cholesterol efflux in HPSC has been also underlined in *LDLR-/-ApoA1+/-* mice that, despite having similar hypercholesterolemia as *LDLR-/-* controls, had diminished HDL cholesterol and greater expansion of HPSC along with neutrophilia and monocytosis. A clinical trial study in children with familial hypercholesterolemia consistently demonstrated increased monocyte counts in children with lower HDL cholesterol compared with those displaying higher HDL-C levels [48]. In vitro enrichment of cholesterol by native LDL or oxLDL treatment of HPSCs has also been shown to promote HPSC proliferation [48]. LXRs have also been shown to promote cholesterol efflux in HPSC s and myeloid progenitor cells, also diminishing their proliferation by reducing their response to IL3 and granulocyte macrophage colony-stimulating factor (GM-CSF) [38,44].

A new mechanism for increased extramedullary HSPC expansion and mobilization in hypercholesterolemic conditions has recently been reported [78]. HPSC was shown to differentiate from hemogenic endothelium through trans-activation of notch embryonic signaling by SREBP2, which attributes a major role to SREBP2 in this cellular type in hematopoiesis rather than cholesterol biosynthesis. Consistent with these findings, the authors reported that hypercholesterolemic Western diet-fed LDLR mice displayed an increase in HSPC, which was abolished by betulin-mediated *Srebf2* inactivation. Moreover, in hypercholesterolemic subjects, LDL cholesterol levels positively correlated with HPSC frequency and were associated with activated SREBP2/Notch signaling HSPC [78].

Quiescence and reconstitution capacity of HPSCs in hypercholesterolemic conditions, through epigenetic regulation, by the key DNA methylation enzyme ten-eleven translocation-1 (TET1), has also been reported. High cholesterol levels, induced by *ApoE*-deficiency or high cholesterol diet, downregulated TET1 gene expression in HPSCs. In agreement with this, *Tet1-/-* deficiency increased total HPSC but decreased HSPC subpopulation with reconstituted stem cell capacity and induced aging characteristics [79]. The mechanism of *TET1* deficiency augmented cell cycle inhibitors p19 and p21 through histone H3K27me3 modification and induced telomere shortening. HPSCs from *Tet1-/-* mice that expressed the murine catalytic domain of TET1 restored telomere length and reconstitution capacity when transplanted. This study demonstrates that TET1 plays a critical role in maintaining the quiescence and reconstitution capacity of HPSCs and that hypercholesterolemia accelerates the HPSC aging phenotype by decreasing TET1 expression.

## 5. Conclusions

Disrupted circulating cholesterol levels provoked by disturbed lipid metabolism is one of the main risk factors for atherosclerosis development and CVD, and consequently, the restoration of lipid homeostasis through different therapeutic strategies has been the main goal to reduce CVD incidence. Notwithstanding this, patients on intensive therapy treatment for CVD display additional residual inflammatory risk. In the last few decades, many studies have shown that cholesterol handling of immune cells has a major impact on lesion progression by modulating their activity and function independently of circulating cholesterol levels or cholesterol hepatic control. In macrophages, cholesterol deposition promotes foam cell formation and predisposes to a proinflammatory phenotype with inflammasome activation and downregulation of LXR-dependent mechanisms. In neutrophils, cholesterol accumulation facilitates NETosis and events associated with atheroma plaque instability. Defective efflux of cholesterol in T cells promotes clustering of TCR and cytokine components in lipid rafts, leading to augmented T cell signaling, activation, and effector Th cell differentiation. Enhanced cholesterol in lipid rafts in HPSC also induces proliferation and mobilization from BM as well as neutrophilia and monocytosis. Taken together, these findings indicate that a better understanding of intracellular handling and effects of cholesterol in immune cells and associated immunometabolism might help reduce atherosclerosis complications.

## Abbreviations

ABCA1	adenosine triphosphate-binding cassette transporter A1
ABCB11	adenosine triphosphate-binding cassette subfamily B member 11
ABCG1	adenosine triphosphate-binding cassette transporter G1
ABCG8	adenosine triphosphate-binding cassette transporter G8
ABCG5	adenosine triphosphate-binding cassette transporter G5
Acetyl-CoA	acetyl coenzyme A
ACAT	acetyl-CoA acetyltransferase
ApoE	apolipoprotein E
BM	bone marrow
CETP	cholesteryl ester transfer protein
CRP	c-reactive protein
CV	cardiovascular
CVD	cardiovascular disease
CYP7A1	cytochrome P450 family 7 subfamily A member 1
FXR	farnesoid X receptor
G-CSF	granulocyte-colony stimulating factor
GM-CSF	granulocyte-macrophage colony-stimulating factor
HDL	high-density lipoprotein
HFD	high-fat diet
HL	hepatic lipase
HMG-CoAR	3-hydroxy-3-methyl-glutaryl coenzyme A reductase
HNF	hepatocyte nuclear factor
HSPC	hematopoietic stem and progenitor cell
IDL	intermediate-density lipoprotein
INSIG1	insulin-induced gene 1
LDL	low-density lipoprotein
LDL-C	low-density lipoprotein cholesterol
LDLr	low-density lipoprotein receptor
LPL	lipoprotein lipase
LPS	lipopolysaccharide
LSK+	Lin-Sca+cKit+
LXR	liver X receptor
MPO	myeloperoxidase
NCP1L1	Niemann–Pick C1-Like 1
NE	neutrophil elastase
NETs	neutrophil extracellular traps
NF-κb	nuclear factor kappa B
NF-Y	nuclear transcription factor Y subunit alpha
PCSK9	proprotein convertase subtilisin kexin 9
PPAR	peroxisome proliferator-activated receptor
PXR	pregnane X receptor
ROS	reactive oxidant species
RXR	retinoid X receptor
SCAP	SREBP cleavage activating protein
SP1	site 1 protease
SQLE	squalene monooxygenase
SRA	scavenger receptor type A
SRBI	scavenger receptor class B type 1
SRE	sterol regulatory element
SREBP2	sterol regulatory element-binding protein 2
SSD	sterol-sensing domains
TCR	T cell receptor
TET1	ten-eleven translocation-1
Th	T helper
TLR	toll-like receptor
Treg	regulatory T cells
VLDL	very low density lipoprotein
VSMCs	vascular smooth muscle cells
